# Spontaneous renal cyst rupture in a female patient: A case report

**DOI:** 10.1016/j.ijscr.2022.107614

**Published:** 2022-09-07

**Authors:** Ronald Sugianto, Pande Made Wisnu Tirtayasa, Alwyn Geraldine Samuel, Maria Yoanita Astriani, Mahendro Aji Panuntun

**Affiliations:** a Faculty of Medicine, Universitas Udayana, Bali, Indonesia; b Department of Urology, Faculty of Medicine, Universitas Udayana, Bali, Indonesia; c Department of Surgery, Tarakan Regional Hospital, Jakarta, Indonesia; d Department of Radiology, Tarakan Regional Hospital, Jakarta, Indonesia

**Keywords:** Renal cyst, Subcapsular hematoma, Spontaneous rupture, Nephrorrhaphy, Case report

## Abstract

**Introduction and importance:**

Spontaneous renal cyst rupture is a rare disease process. Renal masses are heterogeneous tumors that can be benign masses to cancers. This case report aims to provide a brief overview of the renal cyst, clinical symptoms, and management considerations for similar cases.

**Case presentation:**

A previously healthy 30-year-old complained of left back pain a few days ago. There were no abnormalities in the physical and laboratory examination, so CT Scan Abdomen with contrast was performed. It was found that there was a 7.4 cm × 7.0 cm × 7.0 cm cyst. The patients undergo conservative management. Three months later, the patient suddenly fell with severe left back pain. Due to suspicion of spontaneous rupture of the renal cyst, the patient underwent exploration and bleeding control.

**Clinical discussion:**

The advice to wear an abdominal corset is mandatory to protect against the renal cyst. Atraumatic renal hemorrhage has been associated with a classic Lenk's triad (hypovolemic shock, flank mass, and severe flank pain). The definitive management of renal cyst rupture is initiated by resuscitation, followed by an angiographic embolization or surgical management.

**Conclusion:**

The conservative management should be accompanied by advice to use an abdominal corset to protect the left flank from unintentional pressure.

## Highlights

• Spontaneous renal cyst rupture is a rare disease process.
• Hyperdense lesion accompanied by subcapsular renal hematoma
• Renal cyst underwent conservative management
• Exploration and bleeding control showed an active blood vessel that burst.

## 1 Introduction and importance

Spontaneous renal cyst rupture is a rare disease process. Renal masses are heterogeneous tumors that can be benign masses to cancers. Over the past three decades, the incidence rates of renal masses increased dramatically due to commonly axial imaging uses and longer life expectancies [1]. The renal masses classification based on CT-Scan was introduced 30 years ago by Bosniak. The Bosniak classification divided renal masses into I - IV classes, in which infection, inflammatory, and vascular etiologies are excluded. After 25 years of observation, Bosniak summarized that class I masses were simple cysts, class II masses were more complicated but benign, and both categories were benign masses, but class IIF were worrisome enough to be followed up. The Bosniak class III masses were a combination of both benign and malignant lesions, and class IV masses were malignant lesions [2].

In this report, we discuss a case of a 30-year-old woman who complained of left back pain who diagnosed with a left renal cyst accompanied by subcapsular renal hematoma. While the patient underwent conservative management, the renal cyst was ruptured spontaneously without prior history of strenuous activity and trauma. This case report aims to provide a brief overview of the renal cyst, clinical symptoms, and management considerations for similar cases.

## 2 Case presentation

A 30-year-old woman, a nurse, came to the doctor's clinic complaining of left back pain for seven days in June 2021. The patient had no past illness or urological problems history, The family history for urological problems and routine drug consumption was denied. The vital signs were within normal limits, and the physical examination demonstrated normal results, which were no referred pain, mass, or lesions. The patient underwent routine laboratory tests, including CBC, Kidney Function, Liver Function, Urinalysis, and Urine sedimentation, and showed no abnormal results. Therefore, radiologic imaging was carried out for the diagnosis. The USG abdomen showed a cyst on the left kidney. Based on those data, conservative management was the best option for the patient.

**Fig. 1:**
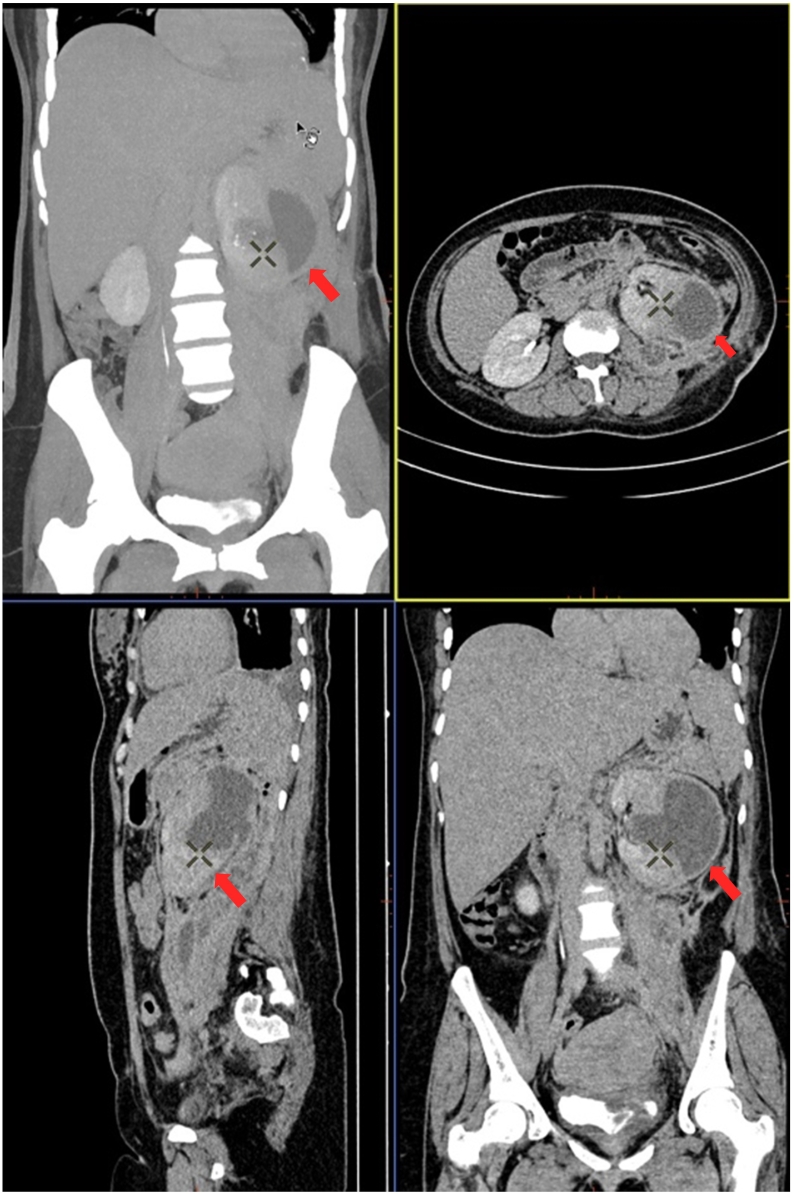
CT SCAN abdomen with contrast showed (red arrow) hyperdense lesion, size ± 7.4 × 7.0 × 7.0 cm, appears to compress the left renal pelvis. (For interpretation of the references to colour in this figure legend, the reader is referred to the web version of this article.)

**Fig. 2:**
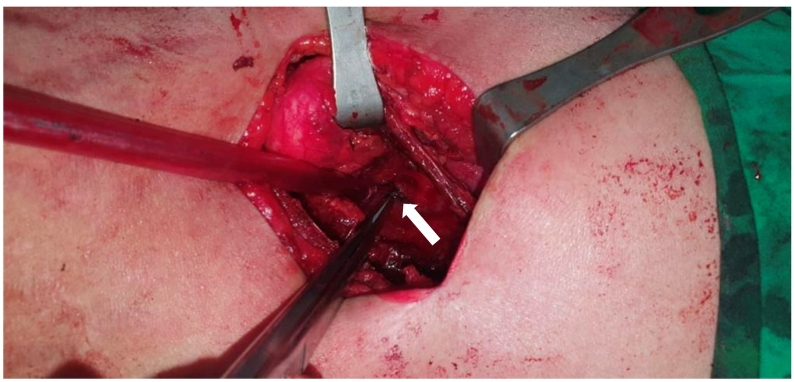
The exploration and bleeding control used cyst excision and nephrorrhaphy technique. The white arrow showed the bleeding control of the burst vessel on the base cyst.

In July 2021, the patient suddenly felt accompanied by severe pain in the left flank region while carrying her baby. The patient denied any history of strenuous activity and trauma. The patient appeared pale and weak, and her vital sign showed low blood pressure and tachycardia. The laboratory examination results demonstrated anemia and low hematocrit level. The results of a CT-Scan abdomen with contrast showed that there was a hyperdense lesion, a well-defined, regular edge on the left kidney, size ± 7.4 × 7.0 × 7.0 cm, with a calcified component, the lesion appears to compress the left renal pelvis, accompanied by subcapsular fluid collection, density 32 Hounsfield Unit (HU) and gloom in the left fat mesentery reaching the region left lower hemiabdomen (Fig. 1). Other findings in the patient included ascites in the pelvic and *peri* splenic region, bilateral pleural effusion, especially left, hepatomegaly, and thickening of the VU wall. Due to suspicion of spontaneous rupture of the renal cyst, the patient underwent exploration and bleeding control. The exploration results obtained that on the base cyst, there was an active blood vessel that bursts. The operative technique for this condition through cyst excision and nephrorrhaphy was performed by Tarakan Regional Hospital Urologist (Fig. 2). Moreover, pathological anatomy analysis demonstrated no malignancy cells found.

As a comprehensive treatment, ensuring any abnormality in the following days was mandatory. The patient underwent a routine follow-up for perioperative, one-week post-operative, and a month later. The patient's daily routine had no limitations, without any post-operative adverse effects. This case presentation followed SCARE Guideline Checklist 2020 [3].

## 3 Clinical discussion

The Bosniak classifications render specific recommendations, Bosniak I and II were managed conservatively, Bosniak IIF should be followed up in more detail, and Bosniak III and IV masses have to be evaluated for other abnormalities findings and management choices [2]. According to AUA guidelines, cross-sectional abdominal imaging, high-quality and multiphase, should be done in patients with a solid or Bosniak III/IV complex cystic renal mass to assess the tumor complexity, degree of contrast enhancement, and presence of fat [1]. Our patient was categorized as Bosniak II masses and underwent conservative management. The advised to wear an abdominal corset are mandatory to protect the renal cyst in the flank from unintentionally increasing abdominal pressures during activity or coughing [4].

Atraumatic renal hemorrhages are associated with a classic Lenk's triad, including hypovolemic shock, flank mass, and severe flank pain. However, all triads are uncommonly concurred altogether, about 20 % of cases. The most frequent symptoms are abdominal and flank pain, 67 % of cases, haematuria, 40 % of cases, and hemorrhagic shock, 26.5 % of cases. In this report, the patient felt severe pain in the left flank region and the signs of hemorrhagic shock, so the patient had two classic symptoms [5]. In imaging studies, the attenuation value of the intra-cystic content will increase approximately 70–90 HU in acute hemorrhage and the attenuation values tend to decrease after blood liquefies [6].

The definitive management of renal cyst rupture is initiated by resuscitation and followed by an angiographic embolization or surgical management. Surgical management is preferred in hemodynamic instability patients due to uncontrolled massive bleeding or malignancy [5], [6]. Nephrorrhaphy is the most frequent technique used to perform renal reconstruction. A partial nephrectomy may be necessary when the renal tissue is unviable. In the lack of possibility to preserve the renal tissue, total nephrectomy should be beneficial [7], [8]. Moreover, nephrectomy is a primary treatment for malignant tumors [5].

The prevention of spontaneous renal cyst rupture while doing conservative management is the recognition of the vulnerable to rupture, including a diameter over 7.5 cm accompanied by infection, strenuous activity, and trauma. Therefore, percutaneous aspiration should be done before the cyst diameter reaches the threshold [4].

## 4 Conclusion

The renal masses, classified as Bosniak I and II, can be managed conservatively. The conservative management includes advice to use an abdominal corset to protect the left flank from unintentional pressure. Aspiration of the renal cyst before had to be considered to prevent the rupture of the renal cyst. Increasing attenuation value is the sign marking of acute hemorrhagic.

## Provenance and peer review

Not commissioned, externally peer-reviewed.

## Funding

None.

## Ethical approval

None.

## Consent

The informed consent was written by the patient in the Indonesian language for further publication of this case report and radiology images anonymously.

Written informed consent was obtained from the patient for publication of this case report and accompanying images. A copy of the written consent is available for review by the Editor-in-Chief of this journal on request.

## Registration of research studies

N/A.

## Guarantor

Ronald Sugianto.

Alwyn Geraldine Samuel.

## CRediT authorship contribution statement

**Ronald Sugianto:** Conceptualization, Formal analysis, Investigation, Methodology, Project administration, Visualization, Writing – original draft, Writing – review & editing. **Pande Made Wisnu Tirtayasa:** Conceptualization, Formal analysis, Funding acquisition, Investigation, Methodology, Project administration, Resources, Supervision, Validation, Visualization. **Alwyn Geraldine Samuel:** Conceptualization, Data curation, Funding acquisition, Investigation, Resources, Software, Supervision, Validation, Visualization. **Maria Yoanita Astriani:** Conceptualization, Data curation, Funding acquisition, Investigation, Resources, Software, Supervision, Validation, Visualization. **Mahendro Aji Panuntun:** Conceptualization, Data curation, Funding acquisition, Investigation, Resources, Software, Supervision, Validation, Visualization.

## Declaration of competing interest

None.
